# Thrombotic Microangiopathy: Multi-Institutional Review of Pediatric Patients Who Underwent HSCT

**DOI:** 10.3390/jpm11060467

**Published:** 2021-05-25

**Authors:** Archana Ramgopal, Shiva Sridar, Jignesh Dalal, Ramasubramanian Kalpatthi

**Affiliations:** 1 Department of Pediatric Hematology Oncology, UPMC Children’s Hospital of Pittsburgh, Pittsburgh, PA 15224, USA; ram.kalpatthi@chp.edu; 2 Case Western Reserve Rainbow Babies Children’s Hospitals, Cleveland, OH 44106, USA; shivasridar@gmail.com (S.S.); jignesh.dalal@uhhospitals.org (J.D.)

**Keywords:** thrombotic microangiopathy, transplant-associated thrombotic microangiopathy, graft-versus-host disease, hematopoietic stem cell transplant, pediatric

## Abstract

Thrombotic microangiopathy (TMA) is a rare but serious complication of hematopoietic stem cell transplantation (HSCT). The purpose of our study is to estimate the incidence, prevalence, and analyze the risk factors and outcome of TMA in children receiving HSCT. Patients under the age of 21 who underwent HSCT at one of the 42 Pediatric Health Information System (PHIS) hospitals from 2000–2012 were analyzed, including demographics, hospitalizations, TMA, and other HSCT-related complications. From 2000 to 2012, a total of 12,369 unique pediatric patients who received HSCT were identified. Among these, 93 (0.8%) children were identified to have the diagnosis of TMA. TMA was significantly associated with allogeneic HSCT, peripheral blood stem cell trasnplants (PBSCT), cytomegalovirus (CMV), human herpes virus 6 (HHV6), fungal infection, graft-versus-host disease (GVHD), and veno-occlusive disease (VOD) (*p* = 0.01). Multivariate logistic regression analysis of mortality showed only HHV6 was an independent risk factor associated with increased mortality in patients with TMA (hazard ratio: 2.86 [1.01, 8.39], *p* = 0.05). The prevalence of TMA in our study is 0.8% with a mortality in our pediatric TMA cohort of 30%, which is in contrast to the higher mortality reported in previously published, small-case series. HHV6 emerged as not only a risk factor for TMA but also as associated with increased mortality in these patients.

## 1. Introduction

Transplant-associated thrombotic microangiopathy (TA-TMA) is a rare but serious complication of hematopoietic stem cell transplantation (HSCT). As it is considered a part of the family of thrombotic endothelium disorders, the disease causes microangiopathic hemolytic anemia leading to microvascular thrombosis and fibrin deposition in the microvessels. In the pediatric population, our review of the literature shows variable incidence of TMA following HSCT with high mortality. Due to a lack of clear diagnostic criteria, it is difficult to determine incidence and mortality for the disease, but values are estimated to be 3% and 60%, respectively [1]. TA-TMA is highly associated with and thought to be preceded by GVHD, but the mechanism of action is still under much debate. Currently, research shows that the disease is induced by endothelial cell injury, which could be the result of either infection, GVHD, chemotherapy, or radiation. Research also demonstrates that certain patients have a predisposition to the development of TMA due to the activation of certain genes involved in the complement pathway. We are currently working in the paradigm of a three-hit hypothesis, where patients with underlying genetic factors (Hit 1) that undergo conditioning regimens such as chemotherapy and/or radiation (Hit 2) and experience other potential sources of endothelial injury, such as infection, medication, or GVHD symptoms (Hit 3), are more likely to manifest the disease [2]. Regarding incidence of TA-TMA, literature demonstrates increased incidence status post-allogeneic HSCT when compared to its autologous counterpart. Other variables that are known to affect incidence of TA-TMA include the primary disease, gender, conditioning intensity, and medications used [1]. There is, however, very little data regarding infection as a risk factor for TA-TMA. While a few case studies of pediatric patients note that TA-TMA was preceded by a human herpes virus 6 (HHV6) infection, there is an ultimate lack of data regarding HHV6 association with TA-TMA [3,4]. The purpose of our study is to estimate the incidence and prevalence of TA-TMA in children receiving HSCT and reveal other potential risk factors.

## 2. Methods

We used the Pediatric Health Information System (PHIS), an electronic database of children’s hospitals in the US. The study was deemed exempt by the local Institutional Review Board. De-identified patients under the age of 21 who underwent HSCT at one of the 42 PHIS hospitals from 2000–2012 were analyzed using data abstracted with ICD-9 codes. All statistical analyses were performed using SAS software, version 9.3 (SAS Institute, Cary, NC, USA) or the base R statistical package (R Foundation for Statistical Computing, Vienna, Austria). Differences in outcomes between patients with and without TMA were assessed by chi-square tests or Wilcoxon nonparametric test for categorical and continuous variables, respectively. Univariate and multivariate regression models were used to identify risk factors associated with TMA and the association of TMA on outcomes. All calculated *p* values were two-sided, and *p* < 0.05 were considered statistically significant.

## 3. Results

**Figure 1:**
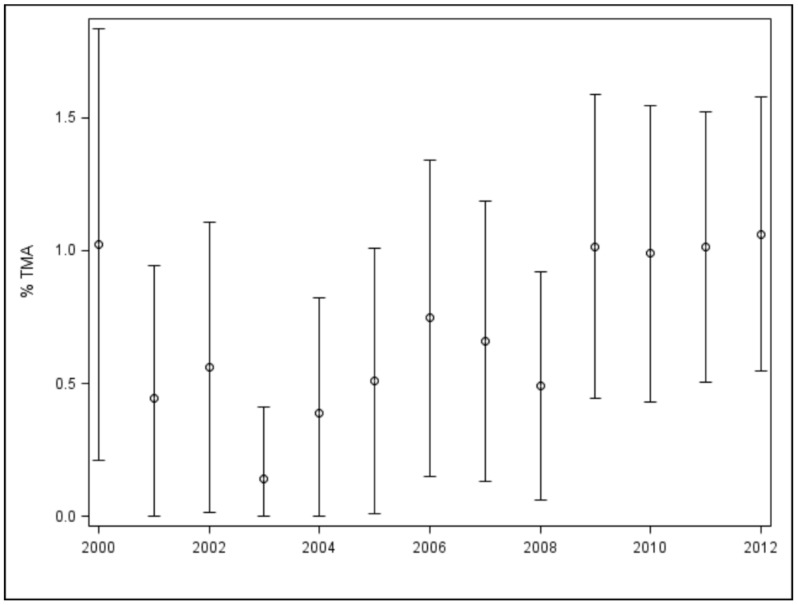
Thrombotic microangiopathy (TMA) trend in pediatric HSCT (hematopoietic stem cell transplantation) recipients, the circles represent average percentages per year with the lines representing the range of percentages.

From 2000 to 2012, a total of 12,369 unique pediatric patients who received HSCT were identified. Among these, 93 (0.8%) children were identified to have the diagnosis of TMA. Overall, there was an increasing trend of TMA diagnoses seen in our cohort over the years, with the highest percent occurrence in 2012 (*p* = 0.0125, Figure 1).

In Table 1, we identify 12 cases of TMA (12.9%) in patients who received an autologous HSCT, 78 cases (83.9%) in patients who received an allogeneic transplant, and 3 cases (3.2%) who were unspecified. Furthermore, 25 cases of TMA (32.1%) were from a bone marrow transplant, 41 cases (52.6%) were after a peripheral blood transplant, and 12 cases (15.4%) occurred after a cord blood transplant. TMA was significantly associated with allogeneic HSCT (*p* ≤ 0.001), PBSCT (*p* = 0.045).

Cytomegalovirus (CMV) (*p* ≤ 0.001), HHV6 (*p* ≤ 0.001), fungal infection (*p* ≤ 0.001), GVHD (*p* ≤ 0.001), and veno-occlusive disease (VOD) (*p* = 0.01).

Regarding demographics, we divided age into the 5 distinct categories of 0–1, 2–4, 5–9, 10–14, and greater than 15 years old but found no significant change in risk for TMA in any age group. Regarding renal involvement, we found significant evidence to support increased association with either early or late TMA. There were only 3 patients during this time period who received eculizumab. There was also no significant change in risk associated with sex or race.

**Figure 2:**
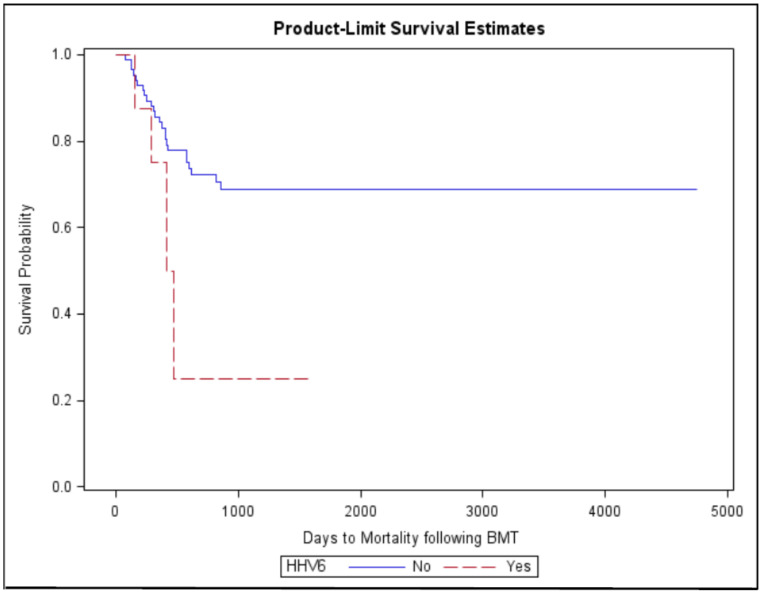
Overall mortality in HSCT patients with human herpes virus 6 (HHV-6).

**Figure 3:**
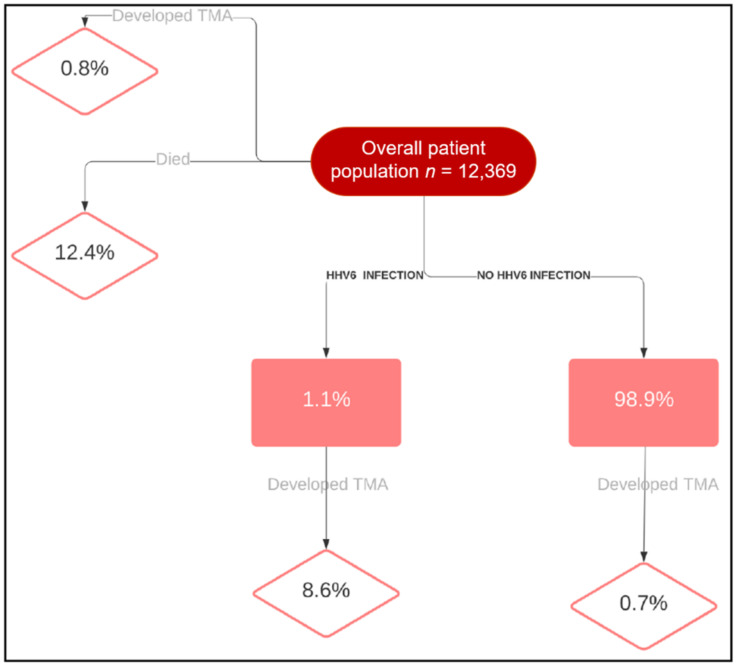
Flow chart of patient study group and the population with HHV6 who developed TMA.

Additionally, TMA was significantly associated with hypertension (*p* ≤ 0.001) and renal failure (*p* ≤ 0.001) (Table 1). In 28 cases of TMA (30.1%), patients required plasmapheresis, and in 22 cases (23.7%), hemodialysis was performed, while patients who did not develop TMA received plasmapheresis and hemodialysis in 0.7% and 3% of cases, respectively. Multivariate logistic regression analysis of mortality using age, gender, HSCT type, CMV, HHV6, fungal infection, and plasmapheresis showed HHV6 was an independent risk factor associated with increased mortality in patients with TMA (Hazard Ratio: 2.86 [1.01, 8.39], *p* ≥ 0.05, Figure 2). Figure 3 depicts a flow chart of the study population.

**Figure 4:**
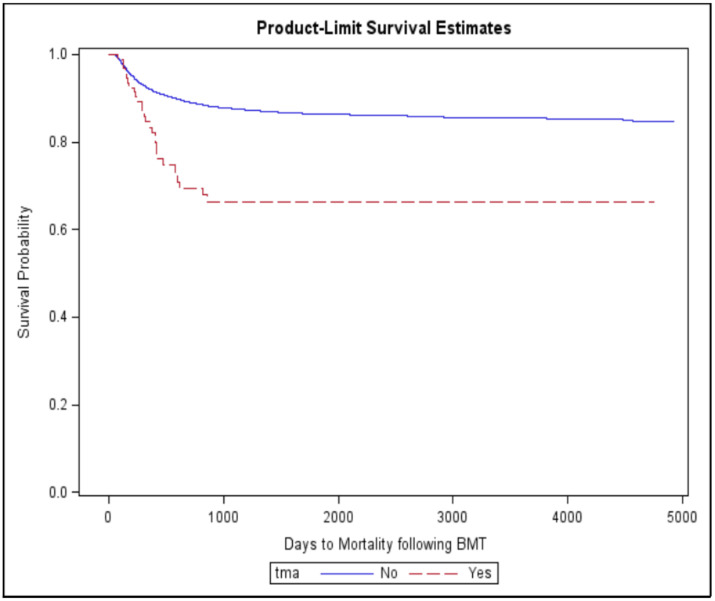
Overall mortality in HSCT patients with TMA.

The mortality was significantly higher in patients with TMA compared to those without (30.1% vs. 12.2%, *p* < 0.001, Figure 4). Additionally, median time (days) to mortality following HSCT was shorter in patients with TMA than those without (754 (365, 1614) vs. 1439 (552, 2847), *p* < 0.0001).

## 4. Discussion

Thrombotic microangiopathy is a severe complication seen following HSCT in children, thought to be associated or preceded by graft-versus-host disease (GVHD). We are currently working in the paradigm of a three-hit hypothesis, where patients with underlying genetic factors (Hit 1) that undergo conditioning regimens such as chemotherapy and/or radiation (Hit 2) and experience other potential sources of endothelial injury such as infection, medication, or GVHD symptoms (Hit 3) are more likely to manifest the disease [5].

Regarding genetic factors, there are several risk factors for TA-TMA that have been identified in the literature. When looking at other microangiopathic disorders, such as atypical HUS, research has shown that many patients present with pathological mutations that promote microangiopathy. These mutations generally either over-promote complement signaling or reduce the available regulators of complement [6]. In the case of TA-TMA, genetic mutations in complement genes have been detected in around 65% of patients. While these mutations are benign in a large percentage of the population, when combined with an endothelial stressor, such as those associated with HCT, they can promote microangiopathic disease [5]. Some studies additionally show that mutations in CD40 ligand and thrombomodulin can predict endothelial complications and mortality after allogeneic stem cell transplantation [7,8]. Three different single nucleotide polymorphisms for the CD 40 ligand were studied, and it was discovered that one, the rs3092936 CC/CT genotype, was associated with an increased risk of TMA and non-relapse mortality following HCT. For thrombomodulin, three out of the seven single nucleotide polymorphisms studied were associated with increased mortality of GVHD.

As demonstrated by previous literature, our data show TA-TMA association with allogeneic HSCT when compared to its autologous counterpart [5,9]. However, our data further distinguished risk among the graft source of HSCT, showing increased TA-TMA association with peripheral blood transplants (PBSCT) compared with bone marrow transplants (BMT). PBSCT are often preferred given that recipients experience a decreased rate of relapse from hematological malignancies and disease-free survival even in patients with end-stage disease [10]. Unfortunately, due to low sample size, we were unable to make any conclusions regarding umbilical cord stem cell transplants. Prior literature has documented increased risk of both acute and chronic GVHD associated with peripheral blood grafts over bone marrow grafts. These studies also led to the conclusion that allogeneic recipients with grade 2 to 4 acute GVHD or viremia should be monitored closely for TA-TMA [11,12]. Additionally, GVHD association with TA-TMA is both well-documented and in agreement with our data. In addition to GVHD, in a study of renal thrombotic microangiopathy after HSCT, TMA was correlated with total body irradiation of >1200 cGy and adenovirus infection [10,13]. We specifically discovered that GVHD had a high association with late TMA, defined as TMA that presents 120 days after transplant, compared to early TMA. Regarding renal involvement, we found no overwhelming evidence to support increased association with either early or late TMA. Although we found a higher incidence of hypertension in late TMA, patients that presented with either early or late TMA required dialysis in similar frequencies.

While it is believed that TA-TMA is endothelial injury-induced, there is much debate regarding the cause of this injury. Firstly, endothelial tissue damage could be associated with GVHD. In chronic GVHD, the endothelium can be damaged as a result of cytotoxic T-cell-induced injury [14]. Other studies have also shown that many of the immunosuppressive agents used to treat GVHD, such as calcineurin inhibitors and mTOR inhibitors, can cause damage to the endothelium [15,16]. Treatments given following transplant, such as radiation and chemotherapy, are also known to be associated with development of TA-TMA [17,18,19].

Our presented data is unique in that it supports yet another pathophysiological mechanism: infection-induced TA-TMA. Specifically, our data show a significant correlation between CMV, HHV6, and fungal infections post-transplant and risk of TA-TMA [20]. Interestingly, we also found that HHV6 infection increases both risk and mortality of TA-TMA. HHV6 is known to be able to directly infect endothelial cells and inhibit angiogenesis [21]. Several studies have noted elevated levels of neutrophil extracellular traps (NETs) in patients with TA-TMA [22]. Mechanistically, it is believed that IL-8 is released by the damaged endothelial tissue, recruiting neutrophils that will undergo netosis and form NETs. These NETs, known up-regulators of complement, then induce complement activation on self-cells, leading to TA-TMA. While normally NETs may be cleared, in patients with chronic GVHD undergoing treatment, the endothelium is constantly being attacked [23]. In patients additionally infected with HHV6, angiogenesis is inhibited, affecting the body’s ability to repair this damage. HHV6 infection may simply play a role in TA-TMA via GVHD, as many studies identify the HHV6 species B as a risk factor for acute GVHD after HSCT [24]. However, it is also of specific interest that the cellular receptor for HHV6B in humans is CD46 or membrane cofactor protein (MCP), a regulator of complement attack on self-cells. In T cells, it is documented that HHV6B infection leads to down-regulation of MCP [25]. Although there is no data on MCP expression on endothelial cells after HHV6B infection, it seems possible that the same finding could hold true for both cell types [26]. If MCP expression is reduced, endothelial cells become more prone to damage via complement attack. While more data are needed to corroborate this claim, HHV6 induced down-regulation of MCP along with promotion of complement signaling via NETs would serve to further expose endothelial cells to damage complement attack and, therefore, promote development and increased mortality of TMA [27].

In the literature, other reports do describe a relationship between a variety of different infectious pathologies and other forms of TMA. Perhaps the oldest and most well-known example is the association of Shiga or Shiga-like exotoxin of *Escherichia coli* with typical hemolytic uremic syndrome (HUS). However, more recently, the relationship between viruses and TMA has been elaborated upon. human immunodeficiency virus (HIV), hepatitis A virus, hepatitis C virus, Dengue virus, rotavirus, norovirus, hepatitis A virus, coxsackievirus, echovirus, influenza A virus, HHV3, HHV4, HHV6, HHV8, CMV, adenovirus, parvovirus and BK virus have all been associated with cases of either thrombotic thrombocytopenic purpura or HUS. In each case, the hypothesized mechanism surrounds the virus’s ability to promote damage to the host’s endothelium [28]. Of particular interest, one case shows BK virus encephalitis in a HCT recipient who developed TMA [29]. It has been suggested in the literature that viruses, such as the influenza virus, can trigger atypical hemolytic uremic syndrome with activation of complement and subsequent TMA. This has been seen not only with all aforementioned viral infections but also recently with coronavirus disease 2019 (COVID-19) infections [30]. Beyond primary lung injury, many COVID-19 patients with moderate to severe infection have displayed significant evidence of coagulopathy. The pathological process appears to be caused by a cascade of thromboinflammation, endothelial injury, and complement activation, leading to TMA. Additionally, some studies show that COVID-19 patients could respond with complement-blocking therapies [31]. Additionally, CMV has also been shown to trigger TA-TMA, as it can cause damage to endothelial cells, causing platelet aggregation through the release of von Willebrand factor. However, the level of quantitative CMV has not been shown to correlate with the degree of allograft engraftment or rejection noted [32].

If possible, it is important to be able to identify patients at risk for TA-TMA prior to initiation of HSCT, which is a study that Higham et al. recently conducted. They used the following criteria to perform risk stratification for patients, and included parameters such as age ≥10 years, HLA mismatch, diagnosis of severe aplastic anemia or malignancy, prior calcineurin exposure, and recipient CMV seropositivity. Additionally, studying the role of prophylaxis is also important, and this study also explored a regimen of eicosapentaenoic acid (EPA), a component of fish oil, with N-acetylcysteine (NAC), given the role these two agents play in maintaining endothelial health. EPA may be beneficial because it has a role in cytokine reduction and production of nitric oxide. NAC removes reactive oxygen species, which can limit damage to the endothelium [33].

Regarding treatment options, there are several clinical trials underway to study the effectiveness of complement-directed therapies earlier in the disease process prior to the development of multi-organ injury. One such therapy is eculizumab, a monoclonal antibody targeted against C5, which has been proven by multiple studies to be associated with higher overall survival compared to untreated controls, in many reports increasing chance survival by over 50% [34]. Similar to eculizumab, ravulizumab is another C5 inhibitor currently in phase 3 clinical trials for the treatment of TA-TMA [35]. Some researchers also propose prophylactic treatment options to limit the third hit in the three-hit hypothesis. Examples include eliminating CNI’s from treatment regimens, increasing antimicrobial prophylaxis to prevent infection induced TMA, and administering rituximab to limit the potential risk of endothelial-directed, antibody-induced TMA and help prevent GVHD [5]. There is also a current phase 2 clinical trial examining the safety of defibrotide, an endothelial-stabilizing agent which could be given prophylactically post-transplant to limit the risk of TMA [36].

Limitations of this study relate to the age of the data collected between 2000 and 2012; however, we believe that our discoveries remain relevant. We did not have conclusive evidence to show any differences between early vs. late TMA or with respect to outcome with eculizumab therapy given the small sample size of patients that received the medication during the study. PHIS is an administrative database; therefore, the patients studied here were identified utilizing ICD-9 codes, which may not fully reflect all complications that ICD-10 codes may cover. Furthermore, additional studies regarding the effect of HHV6 infection on T cells are needed in order to further understand the pathophysiology of virus induced TMA.

## 5. Conclusions

Ours is a large cohort of TMA following HSCT in children. The prevalence of TMA in our study is 0.8% with an increasing trend in recent years. The mortality in our pediatric TMA cohort is 30%, which is in contrast to the higher mortality reported in previously published, small-case series. The literature suggests a three-hit hypothesis, with the first hit being genetic factors, the second hit coming from conditioning regimens such as chemotherapy and/or radiation, and hit three occurring when the patient experiences other sources of endothelial injury, such as medication, GVHD, or infection, such as with viral illnesses. Patients with a genetic mutation in a complement gene, while it may be benign in a large percentage of the population, when combined with a stressor to the endothelium, can promote microangiopathic disease. It will be important moving forward to establish criteria to identify patients at high risk to develop TMA. Our study further distinguished risk among the graft source of HSCT, showing increased TA-TMA association with PBSCT compared with BMT recipients. More recent data will be important to assess the role of therapeutic agents in the prevention and treatment of TMA, such as eculizumab. HHV6 emerged as not only a risk factor for TMA but also as associated with increased mortality in these patients, and CMV was also significantly associated with TMA. Studies exploring the pathophysiology of TMA and its relationship to other complications of HSCT and to prove the effect of HHV6 on complement on a molecular level and therapeutic interventions are needed to optimize the outcome.

## Figures and Tables

**Table 1:** Demographics, risk factors, and outcomes of patients with thrombotic microangiopathy (TMA).

Variable		Overall	No TMA	TMA	*p*
		12,369	12,276	93	
Age	0–1 years	1948 (15.7)	1939 (99.5)	9 (0.5)	0.109
	2–4 years	2819 (22.8)	2793 (99.1)	26 (0.9)	
	5–9 years	2775 (22.4)	2758 (99.4)	17 (0.6)	
	10–14 years	2339 (18.9)	2314 (98.9)	25 (1.1)	
	≥15 years	2488 (20.1)	2472 (99.4)	16 (0.6)	
Sex	Male	7239 (58.5)	7187 (99.3)	52 (0.7)	0.608
	Female	5130 (41.5)	5089 (99.2)	41 (0.8)	
Race	Non-Hispanic White	6974 (56.4)	6914 (99.1)	60 (0.9)	0.265
	Non-Hispanic Black	1450 (11.7)	1445 (99.7)	5 (0.3)	
	Hispanic	2304 (18.6)	2290 (99.4)	14 (0.6)	
	Asian	432 (3.5)	428 (99.1)	4 (0.9)	
	Other	1209 (9.8)	1199 (99.2)	10 (0.8)	
	Other	7560 (61.1)	7494 (99.1)	66 (0.9)	
HSCT Type	Autologous	4043 (32.7)	4031 (32.8)	12 (12.9)	<0.001
	Allogeneic	8177 (66.1)	8099 (66)	78 (83.9)	
	Not specified	149 (1.2)	146 (1.2)	3 (3.2)	
Allo Source	Bone Marrow	3517 (43)	3492 (43.1)	25 (32.1)	0.045
	Peripheral blood	3195 (39)	3154 (38.9)	41 (52.6)	
	Cord blood	1475 (18)	1463 (18)	12 (15.4)	
CMV		958 (7.7)	940 (7.7)	18 (19.4)	<0.001
HHV6		138 (1.1)	130 (1.1)	8 (8.6)	<0.001
Fungal infection		835 (6.8)	814 (6.6)	21 (22.6)	<0.001
GVHD		1471 (11.9)	1443 (11.8)	28 (30.1)	<0.001
VOD		227 (1.8)	222 (1.8)	5 (5.4)	0.011
Hypertension		3288 (26.6)	3226 (26.3)	62 (66.7)	<0.001
Renal failure		1280 (10.3)	1232 (10)	48 (51.6)	<0.001
Plasmapheresis		119 (1)	91 (0.7)	28 (30.1)	<0.001
Hemodialysis		385 (3.1)	363 (3)	22 (23.7)	<0.001
Died		1531 (12.4)	1503 (12.2)	28 (30.1)	<0.001

## Abbreviation

TMA	Thrombotic microangiopathy
HSCT	Hematopoietic stem cell transplantation
GVHD	Graft-versus-host disease
TATMA	Transplant-associated thrombotic microangiopathy
PHIS	Pediatric Health Information System
HHV6	Human herpes virus 6
CMV	Cytomegalovirus

## References

[1] Jodele S., Davies S.M., Lane A., Khoury J., Dandoy C., Goebel J., Myers K., Grimley M., Bleesing J., El-Bietar J. (2014). Diagnostic and risk criteria for HSCT-associated thrombotic microangiopathy: A study in children and young adults. Blood.

[2] Schoettler M., Lehmann L.E., Margossian S., Lee M., Kean L.S., Kao P.C., Ma C., Duncan C.N. (2020). Risk factors for transplant-associated thrombotic microangiopathy and mortality in a pediatric cohort. Blood Adv..

[3] Epperla N., Li A., Logan B., Fretham C., Chhabra S., Aljurf M., Chee L., Copelan E., Freytes C.O., Hematti P. (2020). Incidence, Risk Factors for and Outcomes of Transplant-Associated Thrombotic Microangiopathy. Br. J. Haematol..

[4] Davies J.O.J., Hart A.C., de la Fuente J., Bain B.J. (2018). Macrophage activation syndrome and post-transplant microangiopathy following haploidentical bone marrow transplantation for sickle cell anemia. Am. J Hematol..

[5] Dvorak C.C., Higham C., Shimano K.A. (2019). Transplant-Associated Thrombotic Microangiopathy in Pediatric Hematopoietic Cell Transplant Recipients: A Practical Approach to Diagnosis and Management. Front. Pediatr..

[6] Nester C.M., Barbour T., de Cordoba S.R., Dragon-Durey M.A., Fremeaux-Bacchi V., Goodship T.H., Kavanagh D., Noris M., Pickering M., Sanchez-Corral P. (2015). Atypical aHUS: State of the art. Mol. Immunol..

[7] Rachakonda S.P., Dai H., Penack O., Blau O., Blau I.W., Radujkovic A., Müller-Tidow C., Kumar R., Dreger P., Luft T. (2018). Single Nucleotide Polymorphisms in CD40L Predict Endothelial Complications and Mortality After Allogeneic Stem-Cell Transplantation. J. Clin. Oncol..

[8] Rachakonda S.P., Penack O., Dietrich S., Blau O., Blau I.W., Radujkovic A., Ho A.D., Uharek L., Dreger P., Kumar R. (2014). Single-Nucleotide Polymorphisms Within the Thrombomodulin Gene (THBD) Predict Mortality in Patients with Graft-Versus-Host Disease. J. Clin. Oncol..

[9] Matsuda Y., Hara J., Miyoshi H., Osugi Y., Fujisaki H., Takai K., Ohta H., Tanaka-Taya K., Yamanishi K., Okada S. (1999). Thrombotic microangiopathy associated with reactivation of human herpesvirus-6 following high-dose chemotherapy with autologous bone marrow transplantation in young children. Bone Marrow Transplant..

[10] Stem Cell Trialists’ Collaborative Group (2005). Allogeneic peripheral blood stem-cell compared with bone marrow transplantation in the management of hematologic malignancies: An individual patient data meta-analysis of nine randomized trials. J. Clin. Oncol..

[11] Ye Y., Zheng W., Wang J., Hu Y., Luo Y., Tan Y., Shi J., Zhang M., Huang H. (2017). Risk and prognostic factors of transplantation-associated thrombotic microangiopathy in allogeneic haematopoietic stem cell transplantation: A nested case control study. Hematol. Oncol..

[12] Sakellari I., Gavriilaki E., Boussiou Z., Batsis I., Mallouri D., Constantinou V., Kaloyannidis K., Yannaki E., Bamihas G., Anagnostopoulos A. (2017). Transplant-associated thrombotic microangiopathy: An unresolved complication of unrelated allogeneic transplant for hematologic diseases. Hematol. Oncol..

[13] Changsirikulchai S., Myerson D., Guthrie K.A., McDonald G.B., Alpers C.E., Hingorani S.R. (2009). Renal thrombotic microangiopathy after hematopoietic cell transplant: Role of GVHD in pathogenesis. Clin. J. Am. Soc. Nephrol..

[14] Keesler D.A., St Martin A., Bonfim C., Seber A., Zhang M.-J., Eapen M. (2018). Bone Marrow versus Peripheral Blood from Unrelated Donors for Children and Adolescents with Acute Leukemia. Biol. Blood Marrow Transplant..

[15] Gavriilaki E., Sakellari I., Anagnostopoulos A., Brodsky R.A. (2017). Transplant-associated thrombotic microangiopathy: Opening Pandora’s box. Bone Marrow Transplant..

[16] Heybeli C., Sridharan M., Alkhateeb H.B., Villasboas Bisneto J.C., Buadi F.K., Chen D., Dingli D., Dispenzieri A., Gertz M.A., Go R.S. (2020). Characteristics of late transplant-associated thrombotic microangiopathy in patients who underwent allogeneic hematopoietic stem cell transplantation. Am. J. Hematol..

[17] Biedermann B.C., Sahner S., Gregor M., Tsakiris D.A., Jeanneret C., Pober J.S., Gratwohl A. (2002). Endothelial injury mediated by cytotoxic T lymphocytes and loss of microvessels in chronic graft versus host disease. Lancet.

[18] Luft T., Dietrich S., Falk C., Conzelmann M., Hess M., Benner A., Neumann F., Isermann B., Hegenbart U., Ho A.D. (2011). Steroid-refractory GVHD: T-cell attack within a vulnerable endothelial system. Blood.

[19] Garcia-Maldonado M., Kaufman C.E., Comp P.C. (1991). Decrease in endothelial cell-dependent protein C activation induced by thrombomodulin by treatment with cyclosporine. Transplantation.

[20] Uderzo C., Bonanomi S., Busca A., Renoldi M., Ferrari P., Iacobelli M., Morreale G., Lanino E., Annaloro C., Volpe A.D. (2006). Risk factors and severe outcome in thrombotic microangiopathy after allogeneic hematopoietic stem cell transplantation. Transplantation.

[21] Hoorn C.M., Wagner J.G., Petry T.W., Roth R.A. (1995). Toxicity of mitomycin C toward cultured pulmonary artery endothelium. Toxicol. Appl. Pharmacol..

[22] Kohn S., Fradis M., Podoshin L., Ben-David J., Zidan J., Robinson E. (1997). Endothelial injury of capillaries in the stria vascularis of guinea pigs treated with cisplatin and gentamicin. Ultrastruct. Pathol..

[23] Takatsuka H., Wakae T., Mori A., Okada M., Fujimori Y., Takemoto Y., Okamoto T., Kanamaru A., Kakishita E. (2003). Endothelial damage caused by cytomegalovirus and human herpesvirus-6. Bone Marrow Transplant..

[24] Phan T.L., Carlin K., Ljungman P., Politikos I., Boussiotis V., Boeckh M., Shaffer M.L., Zerr D.M. (2018). Human Herpesvirus-6B Reactivation Is a Risk Factor for Grades II to IV Acute Graft-versus-Host Disease after Hematopoietic Stem Cell Transplantation: A Systematic Review and Meta-Analysis. Biol. Blood Marrow Transplant..

[25] Santoro F., Kennedy P.E., Locatelli G., Malnati M.S., Berger E.A., Lusso P. (1999). CD46 is a cellular receptor for human herpesvirus 6. Cell.

[26] Zipfel P.F., Misselwitz J., Licht C., Skerka C. (2006). The role of defective complement control in hemolytic uremic syndrome. Semin. Thromb Hemost..

[27] Grivel J.-C., Santoro F., Chen S., Fagá G., Malnati M.S., Ito Y., Margolis L., Lusso P. (2003). Pathogenic effects of human herpesvirus 6 in human lymphoid tissue ex vivo. J. Virol..

[28] Lopes da Silva R. (2011). Viral-associated thrombotic microangiopathies. Hematol. Oncol. Stem Cell Ther..

[29] Lopes da Silva R., Ferreira I., Teixeira G., Cordeiro D., Mafra M., Costa I., Bravo Marques J.M., Abecasis M. (2011). BK virus encephalitis with thrombotic microangiopathy in an allogeneic hematopoietic stem cell transplant recipient. Transpl. Infect. Dis..

[30] Sabulski A., Nehus E.J., Jodele S., Ricci K. (2020). Diagnostic Considerations in H1N1 Influenza-induced Thrombotic Microangiopathy. J. Pediatr. Hematol. Oncol..

[31] Wang X., Sahu K.K., Cerny J. (2021). Coagulopathy, endothelial dysfunction, thrombotic microangiopathy and complement activation: Potential role of complement system inhibition in COVID-19. J. Thromb. Thrombolysis.

[32] Java A., Edwards A., Rossi A., Pandey R., Gaut J., Delos Santos R., Miller B., Klein C., Brennan D. (2015). Cytomegalovirus-induced thrombotic microangiopathy after renal transplant successfully treated with eculizumab: Case report and review of the literature. Transpl. Int..

[33] Higham C.S., Collins G., Shimano K.A., Melton A., Kharbanda S., Winestone L.E., Huang J.N., Dara J., Long-Boyle J.R., Dvorak C.C. (2021). Transplant-associated thrombotic microangiopathy in pediatric patients: Pre-HSCT risk stratification and prophylaxis. Blood Adv..

[34] Jodele S., Dandoy C.E., Lane A., Laskin B.L., Teusink-Cross A., Myers K.C., Wallace G.H., Nelson A., Bleesing J., Chima R.S. (2020). Complement blockade for TA-TMA: Lessons learned from a large pediatric cohort treated with eculizumab. Blood.

[35] Study of Ravulizumab in Pediatric Participants with HSCT-TMA—Full Text View—ClinicalTrials.gov. https://clinicaltrials.gov/ct2/show/NCT04557735.

[36] Defibrotide TMA Prophylaxis Pilot Trial—Full Text View—ClinicalTrials.gov. https://clinicaltrials.gov/ct2/show/NCT03384693.

